# Army Correspondence

**Published:** 1863-08

**Authors:** M. M. Eaton

**Affiliations:** Peoria


Peoria, July 29th, 1863.

*Eds. Chicago Medical Journal:*—Having been sent by this city with the Sanitary Commission to Vicksburg, on the City of Alton, to attend to our wounded during the siege of that stronghold, and having visited Shiloh battle-field on the same mission over a year since, and Perryville, Ky., last fall, I feel it my duty to bear testimony to the efficiency of the Medical Corps of the Army. That they are discourteous to civilians I have never found, either on these battle-fields, or in the hospitals of Louisville, Danville, St. Louis, Mound City, Memphis, or any where else. On the contrary they have shown me every courtesy, and as a rule they are men of high moral tone of character, men who have kind hearts as well as skillful hands, men worthy of better pay and more praise than they have received from some presumptuous nurses, who never before, at one time, saw over three sick together. It is a shame that our army medical men are no better paid. Every Surgeon should rank as Colonel, and when in charge of Brigades or Divisions should rank and receive pay as Brig. General.

Then our best Surgeons would go more freely, and those who are competent would not resign so often. "Conservative Surgery" is now all the rage with army Surgeons, and while they cut unmercifully last year, this year they are a little too loth to use the knife. But so it must be, we mortals are always on one extreme or the other.

The Government has now very ample facilities for treating all who may need medical care, save in very extreme cases. In all the armies of the Northwest I have met graduates of old Rush College, who, I am glad to say, in every case to my knowledge, have given unusual satisfaction. Dr. E. Powell, of Chicago, was recognized by Gen. Grant as a man of unusual ability, and placed in a high position, which but proves that true merit will not go unrewarded even when possessed by so modest and unassuming a man as Dr. Powell. All honor to our noble brethren who have sacrificed the comforts of home and family, and dared the uncertain sway of battles, not for pay, not for honor, but out of the promptings of humanity, in their noble hearts, towards their suffering and helpless fellow men.

M. M. EATON.

